# Supplementary material for: Snail Slime Extracted by a Cruelty Free Method Preserves Viability and Controls Inflammation Occurrence: A Focus on Fibroblasts
Source: Molecules. 2023 Jan 26;28(3):1222. doi: 10.3390/molecules28031222 (PMC9920497; doi:10.3390/molecules28031222)
Supplement: Supplementary file 1 [file molecules-28-01222-s001.zip › molecules-2123149-supplementary.pdf]

Table S1: Technical data sheet of microfiltered snail slime

Origin – origin of the molluscs: from farms in Italy, EU and non-EU following the Disciplinary "Cherasco Snail Breeding Method* ".

**Ingredients (INCI)**	
	Snail Secretion Filtrate, Citric Acid, Sodium Benzoate, Potassium Sorbate.
**Reach**	
Snail Slime	N.D.
Sodium Benzoate	01-2119460683-35-0000
Potassium sorbate	N.D.
Citric Acid F.U.	01-2119457026-42
E 330	
**Quantitative Formula**	
RAW MATERIALS	%
SNAIL SECRETION FILTRATE CITRIC ACID<br>SODIUM BENZOATE<br>POTASSIUM SORBATE	
	A
	G
	0,20
	0,20
WE COMMUNICATE THE RANGE ACCORDING TO THE STATEMENTS FDA CRMCS (Food and Drug Administration Composition Statement): A>50%; 25%<B<=50%; 10%<C<=25%; 5%<D<=10%; 1%<E<=5%; 0,1%<F<=1%; G<=0,02%	
**Organoleptic characteristics**	
Appearence	Partially viscous liquid, transparent to slight opalescent. The appearance of the liquid, due to the peculiarities of the product, is characterized by the presence of viscous material that is defined as a possible natural

Colour<br>Smell	deposit and certifies its quality.		
	Color from light to slightly straw yellow. Natural tendency to darken in case of oxidation.		
	Characteristic acidulous (spiritual aroma).		
*https://www.istitutodielicicoltura.it/en/snail-rules-and-regulations/			
**Chemical - Physical characteristics**			
Solubility	soluble in water, in hydroalcoholic (limited), hydroalcoholic, hydroglyceric and surfactant solutions, insoluble in oils.		
pH	2,60 – 4,00	pH meter	Unità di pH
Density	1,00 – 1,30	Densimeter	g/ml
Refractive index	1335- 1345	Refractometry	
Dry residue (105°C, 1h)	1,4 – 6,0	UNI Iso 5725-5:2004 e ISO 13528:2005	g/100g
Hydroxyproline	0,3 – 0,8	M.I.M. Rev. 0:2016	011216/ H
Collagen	2,0 – 2,6	ISO 3496:1994	% m/m
Protein	0,050 – 0,20	ISO1871:2009	g/100g
Mucopolysaccharides	1,0 – 2,0	Gravimetry	g/100 g
Elastin	0,50 -0,60	J.Chrom. A, 1322, 99, 49-53, 2013	%
Glycolic acid	12520	J.Chrom. A, 1322, 99, 49-53, 2013	mg/kg
Allantoin	527	J.Chrom. A, 1322, 99, 49-53, 2013	mg/kg
Magnesium (Mg)	515	M.I.M 110315/A Rev.0:2015	mg/kg
D-lactic acid	<10,0	M.I.M. 01216/A RREV: 0:2016	mg/kg
L-lactic acid	<10,0	M.I.M. 01216/A RREV: 0:2016	mg/kg
Antiprotease	0,50 – 1,50	M.I.M. 01216/A RREV: 0:2016	mU/l
Manganese (Mn)	<1	M.I.M. 110315/A Rev. 0:2015	mg/kg

Vitamin B1 (Thiamine)	0,70 – 1,0	M.I.M. 010611/A rev. 0:2011	mg/kg
Vitamin A	25 - 35	ISTISAN 96/34 pag. 137	u.i./kg
Vitamin C	0,05 – 0,1	M.I.M. 050911/A Rev. 0:2011	mg/kg
Vitamin E	0,4 – 0,8	ISTISAN 96/34 pag. 137	mg/kg
Vitamin B6	25 - 35	ISTISAN 96/34 pag. 137	mg/kg
**Microbiological characteristics**			
Mesophile aerobic bacteria counter	<10	UNI EN ISO 21149:2017	UFC/g
Yeast counter	<10	UNI EN ISO 1612:2017	UFC/g
Mould counter	<10	UNI EN ISO 1612:2017	UFC/g
Candida albicans	Absent	UNI EN ISO 18416:2016+ec 1-2017	UFC/g
Escherichia coli	Absent	UNI EN ISO 21150:2016	UFC/g
Staphylococcus aureus	Absent	UNI EN ISO 22718:2016	UFC/g
Salmonella in 10 gr Ph.Eur	Absent	Ph. Eur. 2.6.13 curr.ed.	In 10 g
Listeria monocytogenes in 10 gr	Absent	UNI EN ISO 11290-1:2017	In 10 g
Pseudomonas aeruginosa	Absent	UNI EN ISO 22717:2016	UFC/g
**Determination of Heavy Metals**			
Arsenic (As)	<1	ICP-MS	mg/kg
Cadmium (Cd)	<1	ICP-MS	mg/kg
Cobalt (Co)	<1	ICP-MS	mg/kg
Total Chromium	<1	ICP-MS	mg/kg
Mercury (Hg)	<1	ICP-MS	mg/kg

Nichel (Ni)	<1	ICP-MS	mg/kg
Lead (Pb)	<1	ICP-MS	mg/kg
Antimony (Sb)	<1	ICP-MS	mg/kg

**Declaration**	
	We declare that in the raw material called snail slime there are no:<br>Substances C.M.R.<br>NANOMATERIAL Substances O.G.M.<br>BSE / TSE substances IONIZING SUBSTANCES RESIDUAL SOLVENTS (excluding water).<br>We declare that the raw material called snail slime is: NO ANIMAL TESTING
**Allergen**	
	Free from cosmetic allergens (Dir. 2003/15/EC and its amendments).
	Does not contain gluten.
Shelf – life<br>Conservation	18 Months
	The product is stable under the following conditions: away from light and<br>heat sources.<br>Storage slime at ambient temperature.<br>Do not freeze.
Product type	Suitable for cosmetic use.

Snail slime technical data sheet Helix	Emitted the 26/03/2022	Rev.17
Verified, approved	Direction: Lumacheria Italiana s.r.l.	Signature: Quality Department

Snail slime technical data sheet Helix	Emitted the 26/03/2022	Rev.17
Verified, approved	Direction: Lumacheria Italiana s.r.l.	Signature: Quality Department

**How to use – Disposal of the product – Warnings**	
How to use	Handling and use within compliant GMP premises is recommended. Personnel must be qualified. Avoid external

	contamination such as food, drink, smoking. At the end of processing, close the cap firmly and keep as indicated at the point of storage.
Disposal of the product	Snail slime secretion is considered to be a category 3 animal by-product within the meaning of Reg. (EC) 1069/2009. In case of contamination with external agents it is necessary to carry out the disposal according to the category of the contaminant.<br>Do not reuse the original containers. The empty container must be disposed of according to the current standard.
Warnings	Keep in closed containers, away from light and moisture. Exposure to temperatures above 40 °C irreversibly degrades the active components of the product.
**Transport Information**	
	The product is not to be considered dangerous pursuant to the provisions in force on the transport of goods by road (A.D.R.), by rail (R.I.D.), by sea (I.M.D.G.) and by air
**Conclusion**	
	The information contained in this page corresponds to the best of our knowledge at the time of printing. The present document shall be used to provide information on the safe use of the product indicated regarding transport, storage, processing and disposal. The indications are not valid for other products. If the product is mixed with other materials or is processed, the indications contained in the card lose value and it will be the responsibility of the final product manager to develop a new technical and safety data sheet for the new formulation.

Table S2: Methods used for the analysis and characterization of microfiltered snail slime quality.

LUMACHERIA ITALIANA SRL CORSO L. EINAUDI, 40 - 12062 CHERASCO (Cuneo)	**ANNEX 1:**<br>**Snail slime analysis plan for cosmetic use**<br>***Year 2022***<br>Rev 03 - 17/03/2022
Description	***Method***
**TOTAL MICROBIAL COUNT**	
Aerobic Mesoli Bacteria Count	UNI EN ISO 21149:2017
Yeast Counter	UNI EN ISO 16212:2017
Mold Counter	UNI EN ISO 16212:2017
Search for Pseudomonas aeruginosa	UNI EN ISO 22717: 2016
Search for Staphylococcus aureus	UNI EN ISO 22718: 2016
Escerichia coli Research	UNI EN ISO 21150: 2016
Candida albicans Research	UNI EN ISO 18416: 2016+ EC 1-2017
Salmonella research in 10 gr. Ph. Eur	Ph. Eur. 2,6,13 curr. Ed
Search for Listeria monocytogenes in 10g	UNI EN ISO 11290-1:2017
**GENERAL CHEMICAL-ANALYTICAL**	
Aspect	Visible rating
Color	Visible rating
Smell	Visible rating
Solubility	
ph	pH meter
Density	Densimeter
Refractive index	Refractometer
Dry residue (105°C, 1h)	UNI ISO 5725-5:2004 e ISO 13528:2005.
**CHIMICO ANALITICO SPECIFICO – CARATTERIZZAZIONE TOTALE**	
Glycolic acid, titre	J.Chrom. A, 1322, 99.49-53, 2013
Collagen (hydroxy-proline x 8)	ISO 3496: 1994
Allantoin, title	J.Chrom. A, 1322, 99.49-53, 2013
Proteins	ISO1871:2009
Mucopolysaccharides	Gravimetry
Elastin	J.Chrom. A, 1322, 99.49-53, 2013
Magnesium (mg)	M.I.M. 110315/A Rev. 0:2015
D-lactic acid	M.I.M. 01216/A Rev. 0:2016
L-lactic acid	M.I.M. 01216/A Rev. 0:2016
Antiprotease	M.I.M. 01216/A Rev. 0:2016
Manganese (Mn)	M.I.M. 110315/A Rev. 0:2015
Vitamins B1 (Thiamine)	M.I.M. 010611/A Rev. 0:2011
Vitamins A	ISTISAN 96/34 pag. 137
Vitamins C	M.I.M. 050911/A Rev. 0:2011
Vitamins E	ISTISAN 96/34 pag. 137
Vitamins B6	ISTISAN 96/34 pag. 137
**HEAVY METALS ICP-MS DETERMINATIONS 8**	
Arsenic ICP-MS	GC – MS chromatography
Cadmium ICP-MS	GC – MS chromatography

Cobalt ICP-MS	GC – MS chromatography
Chromium ICP-MS	GC – MS chromatography
Mercury ICP-MS	GC – MS chromatography
Nickel ICP-MS	GC – MS chromatography
Lead ICP-MS	GC – MS chromatography
ICP-MS Antimony	GC – MS chromatography
**PESTICIDES**	
Chlorinated pesticides, content	UNI EN 15662:2018
Organo phosphorate pesticides, content	UNI EN 15662:2018
Pyrethroids, content	UNI EN 15662:2018
**TEST OF CONSERVATIVES**	
Potassium sorbate, titre	TMC006 Rev.01 del 07/12/2020
Sodium benzoate, titre	TMC006 Rev.01 del 07/12/2020
